# Emergency department visits in California associated with wildfire PM_2.5_: differing risk across individuals and communities

**DOI:** 10.1088/2752-5309/ad976d

**Published:** 2024-12-11

**Authors:** Jennifer D Stowell, Ian Sue Wing, Yasmin Romitti, Patrick L Kinney, Gregory A Wellenius

**Affiliations:** 1 Department of Environmental Health, Boston University School of Public Health, Boston, MA, United States of America; 2 Center for Climate and Health, Boston University, Boston, MA, United States of America; 3 Department of Earth & Environment, Boston University, Boston, MA, United States of America; 4 Health Effects Institute, Boston, MA, United States of America

**Keywords:** wildfire, climate, emergency department, respiratory, cardiovascular, vulnerability

## Abstract

The threats to human health from wildfires and wildfire smoke (WFS) in the United States (US) are increasing due to continued climate change. A growing body of literature has documented important adverse health effects of WFS exposure, but there is insufficient evidence regarding how risk related to WFS exposure varies across individual or community level characteristics. To address this evidence gap, we utilized a large nationwide database of healthcare utilization claims for emergency department (ED) visits in California across multiple wildfire seasons (May through November, 2012–2019) and quantified the health impacts of fine particulate matter <2.5 *μ*m (PM_2.5_) air pollution attributable to WFS, overall and among subgroups of the population. We aggregated daily counts of ED visits to the level of the Zip Code Tabulation Area (ZCTA) and used a time-stratified case-crossover design and distributed lag non-linear models to estimate the association between WFS and relative risk of ED visits. We further assessed how the association with WFS varied across subgroups defined by age, race, social vulnerability, and residential air conditioning (AC) prevalence. Over a 7 day period, PM_2.5_ from WFS was associated with elevated risk of ED visits for all causes (1.04% (0.32%, 1.71%)), non-accidental causes (2.93% (2.16%, 3.70%)), and respiratory disease (15.17% (12.86%, 17.52%)), but not with ED visits for cardiovascular diseases (1.06% (−1.88%, 4.08%)). Analysis across subgroups revealed potential differences in susceptibility by age, race, and AC prevalence, but not across subgroups defined by ZCTA-level Social Vulnerability Index scores. These results suggest that PM_2.5_ from WFS is associated with higher rates of all cause, non-accidental, and respiratory ED visits with important heterogeneity across certain subgroups. Notably, lower availability of residential AC was associated with higher health risks related to wildfire activity.

## 1. Introduction

Wildfire activity has intensified over the last several decades with notable increases in fire size, frequency, severity, and duration of the wildfire season, especially in the Western United States (US) [1, 2]. In particular, wildfire seasons in California have lengthened due to a combination of higher temperatures, increased severity of droughts, and shifts in precipitation and prevailing wind patterns [3, 4]. These shifts have been partly driven by climate change and are expected to be further exacerbated by future warming.

In addition to the direct physical risks, wildfires pose a crucial threat to public health by exposing large segments of the population to the harmful pollutants found in wildfire smoke (WFS) [5]. As wildfires increase in size and intensity, exposure to WFS will continue to increase, potentially subjecting ever larger populations to particulate matter ⩽2.5 *μ*ms in size (PM_2.5_) at concentrations not typically found in ambient air in the US. Additionally, WFS contains different compositions of PM_2.5_, various pollutants, and chemicals that may enhance its toxicity to human physiological systems [5, 6]. Elevated WFS concentrations across the Northeastern US during the summer of 2023 illustrate how increases in wildfire activity can expose larger populations to harmful WFS-related pollutants, even those notably distant from the wildfires themselves [7]. For this reason, it is critical to continue characterizing the health risks associated with WFS exposures and to identify effective risk reduction strategies.

WFS exposures have been associated with multiple adverse health effects, including respiratory outcomes such as asthma, chronic obstructive pulmonary disorder (COPD), and bronchitis [8–15]. There is less consensus regarding the impacts of WFS on cardiovascular risk, with some studies documenting increased risk of outcomes such as stroke and ischemic heart disease [16–23], and others failing to link exposure to cardiovascular outcomes [6, 24–31]. A further dimension that remains relatively unexplored is the potential disparity in WFS’ health impacts on different segments of the population and across measures of susceptibility, vulnerability, and climate region, which may also have implications for policy and adaptation [32]. While some emerging research has begun to explore the differential effects of WFS by individual and community characteristics, more research is needed to better understand these relationships.

Additionally, differential impacts of WFS exposure by access to air conditioning (AC) remains relatively unexplored in previous research [33, 34]. Examining how AC prevalence potentially modifies the effects of WFS exposure is crucial when assessing related risks. Various AC systems have been proposed as protective measures against multiple environmental hazards, including outdoor air pollution and WFS. This topic warrants particular attention for two key reasons, (1) AC availability often correlates with higher socioeconomic status, potentially reflecting inequities in access to resources for mitigating environmental risks [35], and (2) widespread AC access could serve as an effective strategy for bolstering community resilience against WFS exposure [36, 37]. Understanding these relationships could inform targeted interventions and policies to protect vulnerable populations from the health impacts of WFS, while also addressing potential disparities in exposure and resilience.

This study leverages healthcare utilization claims data on California statewide emergency department (ED) visits during the 2012–2019 wildfire seasons. We extend previous work done by Heft-Neal *et al* [38] and seek to provide additional evidence about how the associations between PM_2.5_ attributable to WFS and risk of ED visits vary across subgroups defined by individual-level and community-level characteristics. Using detailed spatial data on wildfire-specific fine particulate matter (PM_2.5_) concentrations, we first empirically estimate the changes in risk associated with WFS PM_2.5_ exposure for all causes, non-accidental causes, and all respiratory and cardiovascular health outcomes. We then examine the extent to which individual characteristics (age, sex, race), access to residential AC, climate region, and measures of social vulnerability moderate the relationship between exposure to WFS PM_2.5_ and adverse health outcomes.

## 2. Data & methods

The health outcome of interest is change in the incidence rate of ED visits for select causes on each day for the 2012–2019 wildfire seasons across California. Health data were in the form of de-identified patient discharge records obtained from California’s Department of Health Care Access and Information (https://hcai.ca.gov). Records include information on the date of encounter, principal diagnosis claims codes, zip codes of residence, age (in 5 year categories), sex, and race/ethnicity. Our sample includes claims for all ED encounters with a residential zip code located in California during our study period. Principal diagnosis codes were used to identify cause-specific ED based on the 9th and 10th revisions of the International Classification of Diseases (ICD-9 and ICD-10) [39]. We analyzed ED visits for all causes, all non-accidental causes, respiratory and cardiovascular diseases, and pooled outcomes other than cardiovascular or respiratory diseases (see table 1 for definitions). We calculated the number of daily visits and aggregated by cause, age (0–4 years, 5–9 years, 10–14 years, 15–19 years, 20–49 years, 50–74 years, 75+ years), sex (female, male), and race (Asian/Pacific Islander, Black, Hispanic, White, other).

**Table 1.** ED visits to CA hospitals for all-cause and specific outcomes, May–November 2012–2019.

Health outcome	ICD-9 & ICD-10^a^,^b^	ED visits (N)	% Total visits
All causes	001–999, E000–E999, V01–V91	53 402 775	—
	A00–Z99		
Non-accidental causes	001–799	40 366 884	75.6
	A00–R99		
Cardiovascular	390–459	1339 268	2.5
	I1–I99		
Respiratory diseases	460–519	4281 488	8.0
	J00–J99		
Other	001–389, 520–999, E000–E999, V01–V91	47 782 019	89.5
	A00–H95, K00–Z99		

^a^ Diagnosis codes defined by the 9th and 10th revisions of the International Classification of Diseases [39].

^b^ For each outcome, ICD 9 codes appear on the first lines followed by ICD 10 codes on the second lines.

We defined the California wildfire season as May through November to capture months on the year during which most wildfire activity occurs across the state. Our environmental predictor of interest was daily WFS PM_2.5_ concentrations averaged for Zip Code Tabulation Areas (ZCTAs) constructed by Childs *et al* and publicly available [40, 41]. In brief, the Childs *et al* WFS PM_2.5_ was modeled at a 10 km resolution using machine learning methods and a combination of remotely sensed data, ground monitors, and reanalysis datasets. These gridded wildfire PM2.5 data were then aggregated to the ZCTA level using population and area of intersection-weighted averaging. Out-of-sample model performance yielded an *R*^2^ value of 0.67. Statistical controls for weather include gridded mean daily temperature and relative humidity from the North America Land Data Assimilation System (NLDAS, https://ldas.gsfc.nasa.gov/nldas). This publicly available data source includes estimates of meteorology at ∼12 km spatial resolution. Temperature and relative humidity values were extracted from grid cells closest to the ZCTA population centroids and then aggregated to the ZCTA level using population weighting methods to adjust data based on population distribution, similar to those by Spangler *et al* [42].

A key objective of our analysis was to examine whether the risk of ED visits associated with WFS PM_2.5_ is modified by the prevalence of residential AC, social vulnerability, and/or climate region. ZCTA-level residential AC prevalence was estimated by combining information from the American Housing Survey and the American Community Survey with Romitti *et al*’s model of the probability of residential AC ownership as a function of demographic, socioeconomic, housing, and climatic characteristics [43]. Estimates of social vulnerability were obtained from the Social Vulnerability Index (SVI) provided by the Centers for Disease Control and Prevention (CDC) [44]. Briefly, SVI values range from 0 to 1 and are assigned using percentile scores for multiple variables and ranking census tracts relative to others in the same year. These variables are grouped into themes and include socioeconomic data (Theme 1: poverty level, education, per capita income), household composition and disability (Theme 2: age, persons with a disability, single parent households), minority status and language (Theme 3: persons of racial or ethnic minorities and the number of persons who speak English), and housing type and transportation (Theme 4: housing density, mobile homes, households with no vehicle access). AC and SVI data were aggregated to the ZCTA-level using population-weighting methods similar to those described above, which involves population-weighting of census tract centroids closest to the population centers of each ZCTA. AC and SVI categories were defined as very low (<0.20), low (0.20–0.40), moderate (0.40–0.60), high (0.60–0.80), and very high (>0.80), with higher AC scores indicating ZCTA populations with a higher probability of access to cooling and higher SVI scores indicating populations that are more socially vulnerable. We derived California climate region delineations from the California Energy Commission Climate Zone Assignments (www.energy.ca.gov/programs-and-topics/programs/building-energy-efficiency-standards). We assessed potential relationships between AC and SVI-related variables using Pearson’s correlation tests.

We analyzed the associations between daily WFS PM_2.5_ exposure and ED visits using a 1-stage time-stratified case-crossover study design, including aggregating cases by calendar day and ZCTA [45]. Case days were subsequently matched to referent days by ZCTA, calendar month, year, and day of the week. This approach controls for seasonal confounding, unobserved time-invariant geographically heterogeneous influences, and slowly varying potential confounders (e.g. sex, age, race, socioeconomic factors, and neighborhood-level contextual factors) [46]. Estimation was performed by conditional Poisson regression modeling using a distributed lag nonlinear framework [47]. Similar to Heft-Neal *et al*, our main specification used daily lags of PM_2.5_ of up to 7 d before each case and referent day [38]. We modeled the WFS PM_2.5_ exposure-response relationship using natural cubic B-splines with internal knots at the 50th and 90th percentiles of exposure and the lag-response functions using natural cubic B-splines with three knots on the log scale of lags up to 7 d prior to ED visits.

We adjusted all models for lags days 0–7 of mean daily temperature and relative humidity using non-linear exposure-response functions modeled with cubic B-splines with three degrees of freedom. We also controlled for US holidays to account for potential systematic differences in time allocation, outdoor activity, and consequent ambient environmental exposures. The modeled odds ratios are interpreted as risk ratios (RRs, a measure of relative risk) for an individual, and we report the RR and its 95% confidence interval associated with the 95th percentile of lag 0–7 daily WFS PM_2.5_ compared to a counterfactual scenario of no PM_2.5_ from WFS. We repeated these analyses for every 10th percentile of exposure. In addition to estimating the impact of exposure on the relative risk of ED visits in the total population, we fit separate models within subgroups of the population based on the age, sex, and race categories as outlined previously. We further analyzed whether the relative risk of ED visits varied by AC, SVI, SVI theme, and climate region. We also explored changes over time by stratifying on earlier years (2012–2015) and later years (2016–2019).

To test the robustness of our findings, we performed multiple sensitivity analyses. First, we evaluated the utility of including a non-linear term for WFS PM_2.5_ by refitting the model using a linear term for the main exposure. Second, we assessed the importance of meteorological variables by refitting the model, excluding temperature and/or relative humidity. Third, we evaluated the potential for residual confounding by our choice of a metric for temperature, by refitting the model using maximum daily temperatures and temperature percentiles in place of mean daily temperatures. We also conducted select analyses for the entire year to provide context for the season-specific results.

We performed all analyses using R version 4.2.1 and the ‘DLNM’ (version 2.4.7) and ‘survival’ (version 3.3–1) statistical packages. A 2-sided *p*-value of <0.05 was considered statistically significant.

## 3. Results

**Figure 1.**
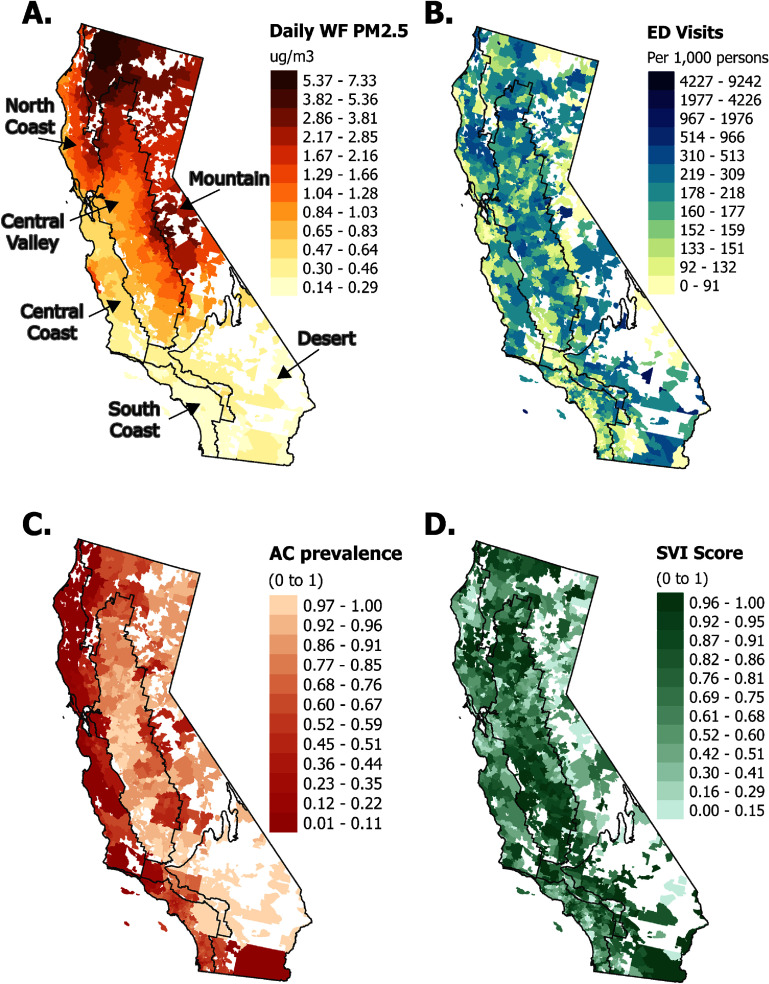
Distribution of seasonal wildfire-specific PM_2.5_, rates of ED visits, AC prevalence, and SVI in California, May to November of 2012–2019. Panel (A): Spatial distribution of daily mean wildfire season (May through November) WFS PM_2.5_ by ZCTA, with the highest concentrations located in central and northern portions of the state. Panel (B): Rates of wildfire season annual emergency department (ED) visits per 1,000 persons by ZCTA, with a range of rates distributed across the state. Panel (C): Prevalence of air conditioning (AC) by ZCTA with lowest AC prevalence along the coast. Panel (D): Distribution of ZCTA-level Social Vulnerability Index (SVI) with the highest vulnerability in the central regions of the state.

The population-weighted mean daily WFS PM_2.5_ concentration at the ZCTA level ranged from 0 to 427.3 *μ*g m^−3^ over the study period, with an overall population-weighted mean of 1.04 *μ*g m^−3^. The highest ZCTA level daily mean WFS PM_2.5_ was observed in Northern California and along the northern Sierra Nevada Mountains in central California (figure 1(A)). Population-weighted mean daily ZCTA level wildfire season temperatures ranged from 12.3 to 108.9 °F and mean daily ZCTA level wildfire season relative humidity ranged from 1.7%–97.2% across the study domain.

We identified over 95 million ED visits in California during our study period. After excluding visits outside the wildfire season and patients without a California zip code, our analytic sample consisted of 50 402 775 records. Rates of ED visits varied by ZCTA throughout the state (figure 1(B)). During the wildfire season, 8.0% of all ED visits had a principal discharge diagnosis related to respiratory and 2.5% to cardiovascular diseases (table 1). Patients were predominantly female (55.0%), aged 20–49 years (42.8%), and Hispanic (39.7%, table 2). Descriptive statistics for the entire time period can be found in tables S1 and S2.

**Table 2.** Number and percent of California ED visits by subgroup May through November 2012–2019.

Description	Subgroup	ED visits (N)	% Total visits
Sex	Female	29 375 406	55.0
	Male	24 025 265	45.0
Age	0–4 years	5176 865	9.7
	5–9 years	2697 635	5.1
	10–14 years	2320 133	4.3
	15–19 years	3328 498	6.2
	20–49 years	22 828 368	42.8
	50–74 years	12 678 684	23.7
	75+ years	4371 834	8.2
Race	White	20 131 910	37.7
	Black	5901 786	11.1
	Hispanic	21 185 332	39.7
	Asian/Pacific Islander	3019 368	5.7
	Other	2488 900	4.7

The prevalence of residential AC is estimated as a percent likelihood of AC ownership and was lowest along the coast of the state (figure 1(C)). As denoted by SVI, social vulnerability was highest in the central portion of the state (figure 1(D)), and distributions of both SVI and AC showed considerable interregional variation (figure S1). The prevalence of AC and the SVI scores were weakly positively correlated. However, AC prevalence showed the strongest correlation with the socioeconomic component of the SVI (Theme 1, includes factors such as poverty, unemployment, income, education, housing cost burden, and lack of health insurance) and the percentage of the population under 18 years of age (figure S2).

**Figure 2.**
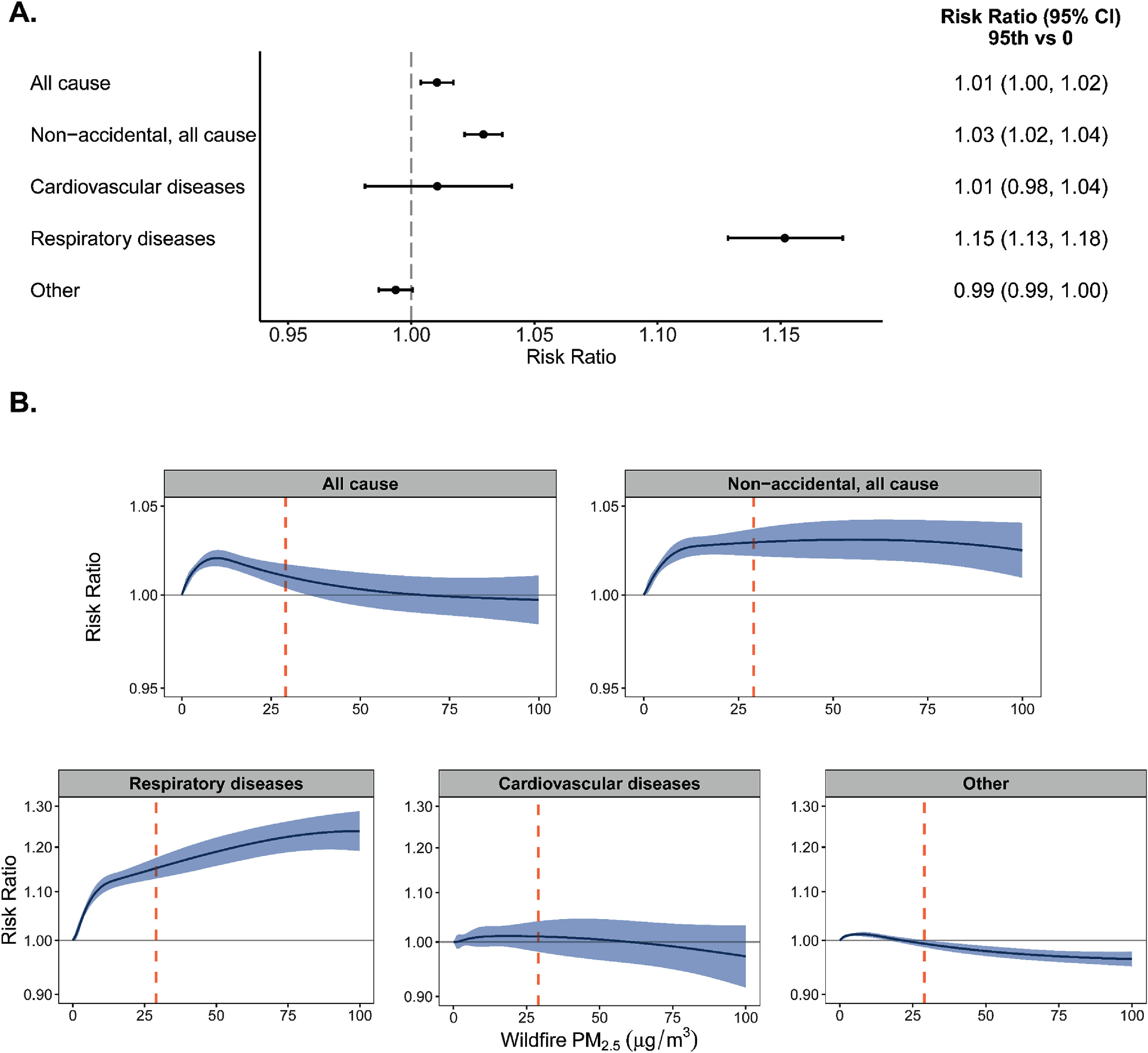
Impact of seasonal wildfire specific PM_2.5_ exposure on ED visits in California, 2012–2019. Panel (A): Risk ratios and 95% confidence intervals of the associations between exposure to wildfire specific PM_2.5_ and emergency department visits comparing the 95th percentile of lag 0–7 exposure to no exposure. Panel (B): Exposure-response curves for the associations between exposure to wildfire specific PM_2.5_ and emergency department visits with 95th percentile in orange.

Our main results are estimated RRs linking cumulative lag 0–7 seasonal WFS PM_2.5_ at the 95th percentile of exposure to all cause, non-accidental all cause, cardiovascular, respiratory, and other ED visits in California from 2012–2019, shown in figures 2 and S3. WFS PM_2.5_ was most strongly associated with ED visits for total respiratory disease (15.17% (12.86%, 17.52%)) and non-accidental all cause (2.93% (2.16%, 3.70%), figure 2(A)). We did not observe a higher relative risk of cardiovascular-related or non-cardiorespiratory ED visits associated with WFS PM_2.5_. Figure 2(B) includes the exposure-response curves for exposures up to 100 *μ*g m^−3^, with the 95th percentile of exposure highlighted in orange for comparison. The risk of ED visits associated with WFS PM_2.5_ for all causes peaked at 10 *μ*g m^−3^, and the risk of non-accidental all cause ED visits associated with WFS PM_2.5_ peaked at 55 *μ*g m^−3^, with increased relative risk at all levels of exposure. The relative risk of respiratory-related ED visits appears to plateau around 100 *μ*g m^−3^. The curves for ED visits for reasons other than cardiorespiratory peaked at 7 *μ*g m^−3^. There was also little evidence of an association between WFS PM_2.5_ and ED visits for cardiovascular disease. Across individual lag days, WFS PM_2.5_ was most strongly associated with the risk of ED visits on lag days 4–7 for all cause and on lag days 0 and 4–7 for non-accidental ED visits (figure S4 and table S3). The relative risk of ED visits for cardiovascular diseases was elevated on lag days 4 and 5, on lag days 0 and 3–7 for respiratory diseases, and on lag days 5–7 for non-cardiorespiratory outcomes.

**Figure 3.**
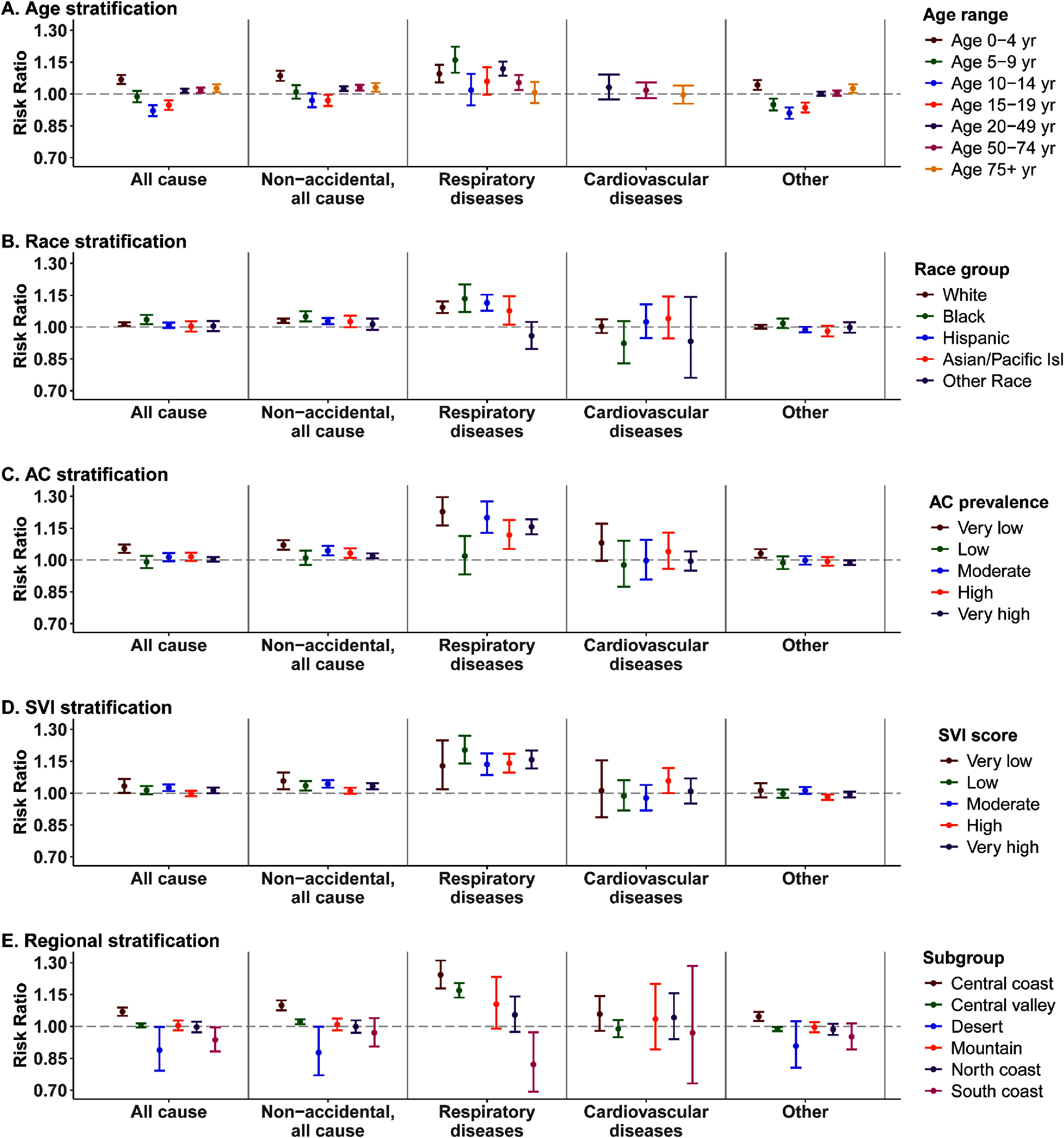
Impact of wildfire specific PM_2.5_ exposure on ED visits by population subgroups in California, 2012–2019. Risk ratios and 95% confidence intervals of the associations between 95th percentile of wildfire specific PM_2.5_ (compared to no exposure) for age groups (Panel (A)), race groups (Panel (B)), air conditioning prevalences (AC, Panel (C)), social vulnerability score (SVI, Panel (D)), and California climate region (Panel (E)). Missing subgroup data points found in some of the panels are due to insufficient data.

The association between WFS PM_2.5_ and the risk of ED visits varied across subgroups of the population as defined by age and race, but not by sex (figures 3(A), (B) and S5). Results by age group for select outcomes are shown in figure 3(A). We observed a higher relative risk of ED visits for all causes and non-accidental causes in children 0–4 years and adults over 20 years compared to children 5–19 years of age. Relative risks of ED visits for overall respiratory diseases tended to be higher in children under ten years of age and in adults 20–74 years old compared to children 11–19 years old. ED visits for non-cardiorespiratory outcomes tended to be higher for children under 4 years of age and in adults over 75 years. When evaluating by subgroups of race, we observed the highest relative risk in the Black population for both all cause ED and non-accidental all cause ED. We also observed elevated risk in the white and Hispanic populations for non-accidental all cause ED (figure 3(B)). The relative risks of ED visits were also highest in the Black population for respiratory diseases and elevated in the white, Hispanic, and Asian/Pacific Islander race groups. No appreciable differences were observed by race for non-cardiorespiratory outcomes or between subgroups defined by sex (figures 3(B) and S5). When stratifying by earlier vs later years, there was a general trend of increasing risk in later years, but the difference was not statistically significant (figure S6).

The relative risk of ED visits generally declined with the increasing prevalence of residential AC, but results were inconsistent (figure 3(C)). The relative risk of all cause ED visits related to WFS PM_2.5_ was statistically significantly associated with the lowest category of AC prevalence. While most categories of AC prevalence showed an increased risk of non-accidental all cause ED visits related to WFS exposure (1.97%–7.00% increased risk), the relative risk was greatest among individuals living in regions with the lowest prevalence of AC. This trend was especially evident for respiratory ED visits, with a 22.75% increased relative risk in the lowest AC stratum. We did not observe an increased relative risk of overall cardiovascular ED visits with any level of AC prevalence.

When stratifying on levels of social vulnerability using the SVI, a meaningful pattern was not evident other than increased relative risk at all SVI levels for overall respiratory disease (figure 3(D)). Some variation was also apparent in an analysis by regions of California (figure 3(E)). For underlying themes of the SVI, the highest risks corresponded with non-accidental and respiratory disease ED visits across all themes (figure S7). The relative risk of all cause and non-accidental all cause ED visits were highest in the Central Coast. The relative risk of non-accidental all cause ED was also increased in the Central Valley region and an increased relative risk of respiratory disease was observed in both the Central Coast and Central Valley regions. No regional differences were observed for cardiovascular diseases or non-cardiorespiratory ED; however, insufficient cases prohibited the evaluation of respiratory and cardiovascular ED visits in the desert region.

Investigation of the region-specific effects of SVI and AC (figures S8 and S9) revealed a higher relative risk of non-accidental all cause ED visits for the lowest AC prevalence category in the South Coast, Central Valley, and Central Coast. The association with WFS PM_2.5_ for respiratory ED visits was also elevated for all categories in the Central Valley and Central Coast regions. When stratifying the SVI score by region, all cause and non-accidental all cause ED visits associated with WFS PM_2.5_ were elevated in the Central Coast, and respiratory ED was elevated for all categories of SVI in the Central Valley and Central Coast.

The results from sensitivity analyses were not materially different from those of the primary analyses for the majority of the observed outcomes (figure S10). Of the model configurations tested, the largest differences were observed with changes in the specification of WFS PM_2.5_ as a linear predictor rather than a nonlinear spline, especially for non-accidental and respiratory ED visits. Additional results using annual models for multiple health outcomes can be found in figures S11–S12.

## 4. Discussion

Despite the growing evidence regarding the potential health harms associated with exposure to WFS, there are still gaps in our understanding of the health effects related to specific subgroups of the population. To address some of these gaps, we leveraged a large health claims dataset from California with over 90 million ED visit records and publicly available exposure datasets and estimated the impact of WFS PM_2.5_ on ED visits for all causes, non-accidental causes, respiratory, and cardiovascular diseases. We also utilize individual characteristics (age, race, sex), modeled AC prevalence, and CDC’s SVI at the ZCTA level to identify modification by subcategories of the population. Our results corroborate current knowledge regarding the association between WFS PM_2.5_ exposure and ED visits for respiratory disease and add to the number of studies showing null or negative associations between exposure and cardiovascular disease outcomes. We also found that the relative risk of ED visits associated with WFS PM2.5 can vary by age and race and that increased relative risk of all cause and non-accidental all cause ED visits was highest in areas with lower AC prevalence.

In comparing our results with those of prior studies, we note that few studies have evaluated the impact of WFS PM_2.5_ on ED visits for all causes and non-accidental causes. Perhaps closest to the present work, Heft-Neal *et al* estimate an aggregate model of weekly ZCTA-level ED visits with location fixed effects on fewer years of California statewide ED data, finding an annual 1.10% increase in the rate of all cause ED visits on days with WFS PM_2.5_ levels between 5 and 15 *μ*g m^−3^ [38]. Interestingly, these authors found a 9.8% reduction in overall ED visits at high WFS PM_2.5_ concentrations, a phenomenon that our results suggest occurs outside the fire season and may be driven by non-respiratory illnesses (figure S11). The latter is also consistent with a hypothesis that wildfires may induce patients to delay treatment of non-WFS related health conditions (figure S12)—perhaps similar to observations noted during the COVID-19 pandemic [48]. We extend the findings of Heft-Neal *et al* by focusing specifically on the wildfire season, including additional years, and using different modeling approaches to understand individual risk of exposure to WFS PM_2.5_. Similar to Heft-Neal *et al*, when using annual data, we observe peak risk increases of ED visits for all causes and non-respiratory causes at 10 *μ*g m^−3^ and 6 *μ*g m^−3^ of exposure, respectively. However, this trend is not seen in ED visits for non-accidental or respiratory outcomes. While more information is needed to, this decline in all cause and non-cardiorespiratory outcomes WFS concentrations increase [49].

Aside from Heft-Neal *et al*, many additional studies rely on smoke day indicators or categories of exposure rather than modeled continuous exposure concentrations. One such study estimated the increase in total ED visits related to smoke days in Sydney, Australia, from 1996–2007 using lags up to 3 d after a smoke event, finding increases in risk for non-accidental ED visits of 1%–3% depending on the specific lag day [19]. Conversely, a study in wildfire-prone Northern California (Shasta County) from 2013 to 2018 found no increase in total ED visits related to a week of high wildfire, defined as ⩾5.5 *μ*g m^−3^ total weekly exposure [50]. Taken together, the range of observed effects appears to be related to study choices, such as the definition of the exposure variable. Here, we estimate a 1.04% and 2.92% increased risk in all cause and non-accidental all cause ED due to lag 0–7 WFS PM_2.5_ exposure, respectively.

There is general consensus regarding the impact of WFS on respiratory disease and multiple prior studies have found associations between WFS PM_2.5_ and various respiratory health outcomes. These studies have investigated health impacts related to physician visits, ED visits, and hospitalizations for respiratory disease [6, 18, 24, 26, 28, 31, 32, 38, 50–57]. For example, one study found a 33% increase in physician visits related to a 10 *μ*g m^−3^ increase in WFS PM_2.5_ exposure [23]. For ED visits, Reid *et al* observed a 3.5% increase in the relative risk of respiratory visits associated with a 10 *μ*g m^−3^ increase in WFS PM_2.5_ [10]. The authors additionally noted an 11.5% increase in the relative risk of asthma ED visits and a 5.4% increase in the relative risk of visits for COPD. In Heaney *et al*, the authors observed a 3% increased risk of respiratory hospitalizations and a 10% increased risk of asthma hospitalizations associated with the 98th percentile of lag 0–1 WFS PM_2.5_ exposure [26]. Our results align with this previous work, showing an increased impact on respiratory healthcare utilization associated with lag 0–7 WFS PM_2.5_ exposure.

While most studies agree on the relationship between exposure and respiratory disease, the relationship between exposure and cardiovascular disease remains uncertain. This uncertainty may be linked to several factors, including exposure misclassification, limited exposure timelines, importance of cumulative exposures, or the use of non-sensitive indicators of cardiovascular morbidity [58]. Various studies have found links between exposure and total cardiovascular disease. However, others have failed to observe a positive link with WFS PM_2.5_ exposure [52]. For example, Chen *et al* observed a 1% increased risk of cardiovascular disease ED in California adults 18–64 years after exposure to a ‘smoke event’, but failed to find a statistically significant increase for any cardiovascular disease category of interest (acute myocardial infarction, cerebrovascular disease, dysrhythmia, ischemic heart disease, and peripheral vascular disease) [52]. Conversely, evaluating the 2017 San Francisco wildfires, Malig *et al* did not observe an increased risk of cardiovascular disease ED visits in time-adjusted models [55]. Our results corroborate the findings of Malig *et al*; however, more research is needed to understand the discrepancies relating to the effect of WFS PM_2.5_ on cardiovascular disease and to identify the factors contributing to this uncertainty.

We observed heterogeneity in the risk of ED visits by exposure level, lag day, and when comparing seasonal to annual results. Identifying the mechanism behind the trends in risk by exposure level is beyond the scope of this paper, however, some of the differences could point to differences with specific outcomes in response to acute exposures and/or changes in health seeking behavior dependent on visible smoke levels or significant smoke warnings. Some of the delay in increased risk by lag day may be related to latency in the development of symptoms or changes in health care seeking behavior. Additionally, the results for the wildfire season differ somewhat from the annual results. Generally, estimated risk using annual data tended to peak and/or decrease at lower levels of exposure. This could be due to lower overall exposure to WFS or, again, to behavioral changes during the non-wildfire season.

Multiple factors may contribute to an individual’s vulnerability to exposure to harmful WFS PM_2.5_ [59–61]. One important factor is access to AC and air filtration. General guidelines for protection during a WFS event include staying indoors and turning on AC and or air purifiers [62]. Utilization of AC and air filtration is generally only feasible if individuals have both access and the monetary means to fund the increased electrical usage associated with their operation [43]. Current research includes very little investigation into the differential impacts of risk by AC prevalence. One study by Do *et al* evaluated AC prevalence as an effect modifier and found a reduction in the risk of a respiratory ED visit related to smoke days with increasing levels of AC [33]. While we found generally similar results for respiratory outcomes, our study reflects increased risk of ED visits for all causes and non-accidental causes in addition to respiratory outcomes and utilizes a distinct WFS dataset. Another clear difference is our use of WFS, exposure as a continuous variable rather than a dichotomous smoke days versus non-smoke days. Finally, our method uses an empirical three-level hierarchical specification to predict ZCTA-level prevalence using census tract level 5 year ACS data (2010–2014 and 2015–2019) aggregated to the ZCTA level.

Our results suggest that individuals living in areas with a lower prevalence of AC may be more vulnerable to ED visits for all causes, non-accidental causes, and respiratory disease. However, this approach has limitations since our modeled AC prevalence only reflects the probability of having residential AC and does not account for actual usage levels. In addition, our results do not consider differences in indoor air filtration or the type of AC unit. While it is true that indoor infiltration of smoke may or may not be affected by AC prevalence, very little is known regarding whether this guidance is protective. It was outside the scope of this current study to determine the types of AC units, structure permeability, and whether AC prevalence translates to usage. However, with the general lack of studies surrounding this topic, we sought to identify whether a difference in risk could actually be discerned using modeled AC prevalence. These nuances likely explain, in part, some of the variability we see in the response (i.e. slight increases in risk for all causes and non-accidental causes for moderate AC prevalence groups). Hence, we merely suggest that there may be some evidence of higher risk in the ‘very low’ AC prevalence group. This warrants further study to test whether this relationship indeed reflects real-world AC usage and effectiveness.

The manner in which populations’ socioeconomic vulnerability modulates WFS PM_2.5_-related ED visit risk is less certain. Our results regarding SVI generally agree with previous literature, however, we did not observe a clear pattern of increasing health risks associated with WFS and vulnerability scores. For example, Reid *et al* did not find meaningful patterns of increased risk with higher social vulnerability. Similarly, we were unable to identify a clear trend, with no clear relationship between increased risk and higher SVI scores. This could be due to the spatial scale used in our analyses or unobserved differences in AC prevalence and its utilization, independent of SVI in California. Romitti *et al* showed a consistent relationship between low AC prevalence and high SVI at the census tract level in 115 US cities, but the strength of this relationship might depend on the geographic scale and exhibit regional disparities [43]. This may be particularly true in the Western US, which has the lowest average rates of AC usage in any US region [63]. California’s unique geography may also be a contributing factor, with large urban areas with low SVI located in relatively mild coastal climates, precluding the need for AC [43].

This study utilizes multiple fire seasons and leverages 50+ million health ED records in California to increase our understanding of the health effects related to WFS PM_2.5_. Our methods employ a high-resolution, publicly available WFS PM_2.5_ model to generate increased relative risk for select cardiorespiratory outcomes and employ methods for estimating AC prevalence at the ZCTA level in California. Despite these strengths, there are some noted limitations to our approach. Our health records are specific to the state of California, and, as such, our results may not be generalizable to populations outside of this state. As with most air pollution health studies, at least some exposure misclassification is unavoidable. This misclassification can be linked to the methods used to estimate the spatially and temporally varying concentrations of WFS and related pollutants, which vary depending on the use of aggregation, interpolation, and weighting methods. Another source of exposure misclassification may be present due to our use of ZCTA of residence to assign exposure. Our study’s retrospective character also limits our ability to understand cases’ mobility and time spent at home versus other locations. Thus, our exposure designation represents probable exposure but does not capture absolute exposure. Additionally, while our health data includes a substantial number of ED records, our analysis does not include hospitalization or outpatient visits. Hence, our results may only represent a portion of the true health effect, especially for outcomes usually seen in outpatient clinics with less frequent ED visits. Finally, the use of modeled AC prevalence in lieu of recorded individual AC prevalence likely affects the actionability of these results. However, as the first study looking at AC and WFS exposure, our results suggest that there may be an inverse relationship between AC and WFS-related health outcomes.

## 5. Conclusions

In summary, this study represents one of the largest studies on the impact of WFS PM_2.5_ across multiple seasons and one of the first to explore AC prevalence in conjunction with susceptibility and social vulnerability. We identified a statistically significant increased relative risk of ED visits for all causes, non-accidental causes, and respiratory outcomes; however, we did not observe a higher relative risk of cardiovascular disease. After stratification, we identified differences in health effects by race, age, and AC prevalence, which warrants further investigation. To better describe the broad health impact of WFS PM_2.5_, it will be important to repeat these analyses for hospitalizations and outpatient clinic visits and to consider other ways to characterize adaptation access and measures of social vulnerability.

## Data Availability

All exposure data is publicly available, and health data may be acquired by contacting California’s Department of Health Care Access and Information (https://hcai.ca.gov). The data that support the findings of this study are openly available at the following URL/DOI: https://doi.org/10.7910/DVN/VHNJBD; https://ldas.gsfc.nasa.gov/nldas.
